# Discovery of TaFeSb-based half-Heuslers with high thermoelectric performance

**DOI:** 10.1038/s41467-018-08223-5

**Published:** 2019-01-17

**Authors:** Hangtian Zhu, Jun Mao, Yuwei Li, Jifeng Sun, Yumei Wang, Qing Zhu, Guannan Li, Qichen Song, Jiawei Zhou, Yuhao Fu, Ran He, Tian Tong, Zihang Liu, Wuyang Ren, Li You, Zhiming Wang, Jun Luo, Andrei Sotnikov, Jiming Bao, Kornelius Nielsch, Gang Chen, David J. Singh, Zhifeng Ren

**Affiliations:** 1 Department of Physics and Texas Center for Superconductivity, University of Houston, Houston, TX 77204 USA; 2 Department of Physics and Astronomy, University of Missouri, Columbia, MO 65211 USA; 3 Beijing National Laboratory for Condensed Matter Physics, Institute of Physics, Chinese Academy of Sciences, P.O. Box 603, Beijing, 100190 China; 4 Department of Materials and Energy, Southwest University, Chongqing, 400715 China; 5 Department of Mechanical Engineering, Massachusetts Institute of Technology, Cambridge, MA 02139 USA; 6 Institute for Metallic Materials, IFW-Dresden, Dresden, 01069 Germany; 7 Department of Electrical and Computer Engineering, University of Houston, Houston, TX 77204 USA; 8 Institute of Fundamental and Frontier Sciences, University of Electronic Science and Technology of China, Chengdu, 610054 China; 9 School of Materials Science and Engineering, Shanghai University, Shanghai, 200444 China

## Abstract

Discovery of thermoelectric materials has long been realized by the Edisonian trial and error approach. However, recent progress in theoretical calculations, including the ability to predict structures of unknown phases along with their thermodynamic stability and functional properties, has enabled the so-called inverse design approach. Compared to the traditional materials discovery, the inverse design approach has the potential to substantially reduce the experimental efforts needed to identify promising compounds with target functionalities. By adopting this approach, here we have discovered several unreported half-Heusler compounds. Among them, the p-type TaFeSb-based half-Heusler demonstrates a record high *ZT* of ~1.52 at 973 K. Additionally, an ultrahigh average *ZT* of ~0.93 between 300 and 973 K is achieved. Such an extraordinary thermoelectric performance is further verified by the heat-to-electricity conversion efficiency measurement and a high efficiency of ~11.4% is obtained. Our work demonstrates that the TaFeSb-based half-Heuslers are highly promising for thermoelectric power generation.

## Abstract (web summary)

The discovery of thermodynamically stable thermoelectric materials for power generation has relied on empirical methods that were not effective. Here, the authors apply the inverse design approach to identify and experimentally realize TaFeSb-based half Heuslers with high thermoelectric performance.

## Introduction

The ever-increasing energy consumption from fossil-fuel combustion has led to alarming environmental impacts. As one of the clean energy conversion techniques, thermoelectric power generation can harvest waste heat and convert it into electricity via the Seebeck effect^1,2^. However, the wide application of thermoelectric power generation systems requires significant improvements in the energy conversion efficiency, which essentially depends on the materials’ dimensionless figure-of-merit (*ZT*). It is defined as *ZT* = *SσT*/(*κ*_lat_+*κ*_ele_), where *S* is the Seebeck coefficient, *σ* is the electrical conductivity, *κ*_lat_ is the lattice thermal conductivity, *κ*_ele_ is the electronic thermal conductivity, and *T* is the absolute temperature^3–7^.

The transport parameters on which *ZT* depends are strongly interrelated with each other due to their different, and typically opposite, dependencies on carrier concentration and electronic structure^8^. Therefore, research on thermoelectric materials has focused on identifying approaches that can effectively decouple the key transport parameters for enhancing the *ZT* of existing materials^9–14^. In the meantime, relentless efforts have also been devoted to discovering promising new compounds that have unusual characteristics enabling favorable combinations of high electrical conductivity, large Seebeck coefficient, and low thermal conductivity. Historically, the materials discovery has mostly relied on the Edisonian trial and error approach. However, such a traditional process becomes increasingly time-consuming. As a result, there is a clear need for more efficient rational approaches to discovering new promising materials. This has provided the motivation for high-throughput methods and other theoretical approaches that use predictions of properties to identify candidate materials^15,16^. Yet a key challenge is to balance the reliability and cost in such studies. A consequence of the low reliability is that the predicted compounds (especially ones that have not yet been experimentally made) with promising thermoelectric performance may be experimentally identified as metastable or unstable. This is a particular challenge for thermoelectrics because a main approach for resolving the opposite dependencies of the transport properties, e.g., *S* and *σ*, is through unusual electronic structures, but unusual electronic properties are often found in calculations for unstable compounds, *e.g*., due to unfavorable bonding. Thus, mere prediction of the thermoelectric properties for the missing compounds is not enough to guarantee experimental success. In fact, analysis of the thermodynamic stability for the unreported compounds and assessment of potential unknown competing phases could provide highly valuable guidance for the experimental efforts. It should be noted that careful experimental synthesis and evaluation of a compound are costly, while most theoretical calculations, especially as applied in high throughput modes, are relatively inexpensive. As such, it might be beneficial to use more sophisticated theoretical studies in predicting compounds before devoting the efforts for careful experimental study.

Recently, Gautier et al.^17^ investigated the thermodynamic stability of 400 unreported half-Heusler compounds and 54 of them were predicted to be stable. Subsequently, 15 compounds were successfully synthesized to verify their prediction. Similarly, Carrete et al.^18^ performed the thermodynamic analysis for 450 half-Heusler compounds and predicted that 77 are stable. In addition, Zakutayev et al.^19^ investigated the thermodynamic stability of the V-IX-IV family (V = V, Nb, and Ta; IX = Co, Rh, and Ir; and IV = C, Si, Ge, Sn, and Pb) of half-Heuslers. Their calculations indicated that among the 29 missing compounds, 8 are stable. TaCoSn, one of those predicted to be stable, was later successfully synthesized, confirming the prediction. Clearly, the inverse design approach^20^, which targets at the specific functionalities and involves with calculations of structure as well as thermodynamic stability and then followed by the experimental realization, is capable of significantly expediting the material discovery.

In our work, we focused on the half-Heusler compounds, which are among the most promising candidates for thermoelectric applications. Thermodynamic stabilities of the V^1^-VIII-V^2^ family (with V^1^ = V, Nb, and Ta; VIII = Fe, Ru, and Os; and V^2^ = As, Sb, and Bi) of half-Heuslers were systematically investigated. As a result, we have discovered 6 undocumented compounds and 5 of them are stable with the half-Heusler crystal structure. The p-type TaFeSb-based half-Heusler, one of the compounds discovered in this work, demonstrated a very promising thermoelectric performance. A peak *ZT* of~1.52 at 973 K and an average *ZT* of ~0.93 in the temperature range of 300~973 K were obtained. The high thermoelectric performance was further verified by the heat-to-electricity conversion efficiency measurement. A record high conversion efficiency of ~11.4% at the cold-side temperature of 317 K and hot-side temperature of 973 K was obtained.

## Results

### Thermodynamic stability analysis

**Fig. 1:**
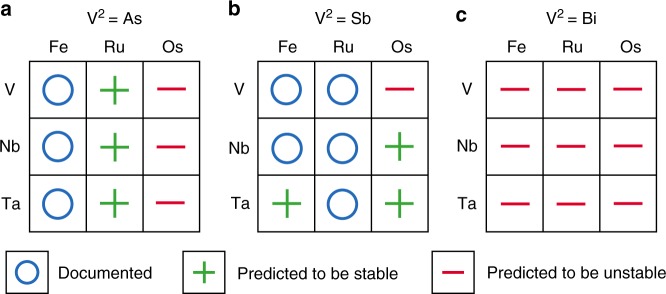
Thermodynamic stability calculation. Calculated stability of the V^1^-VIII-V^2^ family (with V^1^ = V, Nb, and Ta; VIII = Fe, Ru, and Os; and V^2^ = As, Sb, and Bi) of half-Heusler compounds. **a** V^1^-VIII-As, **b** V^1^-VIII-Sb, and **c** V^1^-VIII-Bi

Systematic theoretical calculations on the V^1^-VIII-V^2^ family (with V^1^ = V, Nb, and Ta; VIII = Fe, Ru, and Os; and V^2^ = As, Sb, and Bi) of half-Heusler phases were first conducted. In this family (27 compounds in total), 8 compounds have been documented to be stable while no experimental reports can be found for the remaining 19. Therefore, the crystal structure search for the missing compounds was conducted and the thermodynamic stability analysis of each compound with respect to all of the other competing phases was performed. The key aspect of the crystal structure search is that it allows us to check whether the half-Heusler structure is the true ground state rather than other possibly competing structures with the same 1:1:1 stoichiometry. We predict that 6 compounds (VRuAs, NbRuAs, TaRuAs, TaFeSb, NbOsSb, and TaOsSb) out of the missing 19 are stable, as shown in Fig. 1. All of the predicted stable compounds are in the cubic structure (*F*43*m*), with the exception of NbRuAs that has an orthorhombic structure (*Pnma*). Some of these structures predicted agree quite well with the other theoretical calculations^18,21^. It should be noted that our prediction for NbRuAs differs from that of Carrete et al.^18^. We verified that the orthorhombic structure we found from the crystal structure prediction does have lower energy than the cubic structure they identified. The predicted energy difference is 25 meV per atom. In the following, we will focus mainly on TaFeSb, and the other five stable compounds will be reported elsewhere.

**Fig. 2:**
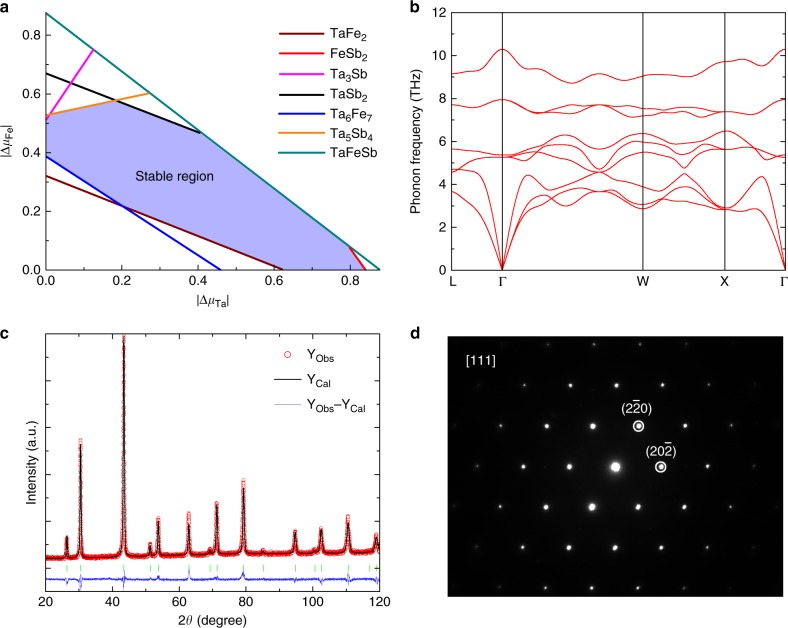
Theoretical prediction and experimental realization of TaFeSb half-Heusler. **a** Phase stability diagram for TaFeSb, where each line represents a known competing phase and the stable region is indicated in violet. It should be noted that $\Delta \mu _{{\mathrm{Fe}}}$ and $\Delta \mu _{{\mathrm{Ta}}}$ are both negative. **b**) Calculated phonon dispersion of TaFeSb. **c** Rietveld refinement for the XRD pattern of the prepared TaFeSb in this work. **d** The [111] zone-axis selected area electron diffraction pattern

For the thermodynamic stability of the V^1^-VIII-V^2^ family, three criteria should be satisfied

1 $$\Delta \mu _{{\mathrm{V}}^1} + \Delta \mu _{{\mathrm{VIII}}} + \Delta \mu _{{\mathrm{V}}^2} = \Delta H_f\left( {{\mathrm{V}}^{\mathrm{1}}{\mathrm{VIIIV}}^{\mathrm{2}}} \right);$$

2 $$\Delta \mu _i < 0,\left( {i = {\mathrm{V}}^1,{\mathrm{VIII}},{\mathrm{V}}^2} \right);$$

and

3 $$\begin{array}{l}n_j\Delta \mu _{{\mathrm{V}}^1} + m_j\Delta \mu _{{\mathrm{VIII}}} + q_j\Delta \mu _{{\mathrm{V}}^2} \le \Delta H_f\left( {{\mathrm{V}}_{n_j}^1{\mathrm{VIII}}_{m_j}{\mathrm{V}}_{q_j}^2} \right),\\ j = 1,...,t,\end{array}$$

where $\Delta \mu _i = \mu _i - \mu _i^0$ is the deviation of actual chemical potential of atomic species *i* during growth (*μ*_*i*_) from that of bulk elemental solid or gas phase ($\mu _i^0$), ∆*H*_*f*_ is the heat of formation, and ${\mathrm{V}}_{n_j}^1{\mathrm{VIII}}_{m_j}{\mathrm{V}}_{q_j}^2$ represents all of the known *j* competing phases, which provide a limitation to the broadening of the stable regions. Eq. (1) is for equilibrium growth, Eq. (2) is to prevent the precipitation of elemental phases of atomic species *i*, and Eq. (3) is to ensure that V^1^-VIII-V^2^ half-Heuslers are stable against the formation of other competing phases. Figure 2a shows the two-dimensional phase stability diagram for TaFeSb with two independent quantities $\Delta \mu _{{\mathrm{Fe}}}$ and $\Delta \mu _{{\mathrm{Ta}}}$. Six competing phases in the Ta-Fe-Sb system have been calculated to evaluate the stability of TaFeSb^22,23^. It should be noted that the Fe-Sb phase diagram includes a Fe_1.27_Sb phase. This phase is characterized by site disorder, and thus was not included in our calculations. A stable region for TaFeSb (marked by violet) exists with the consideration of all known potential competing phases. Such a large stability region for TaFeSb indicates that this compound should be synthesized experimentally. In addition, the lattice dynamical stability of TaFeSb was also examined and its phonon dispersion is shown in Fig. 2b. The absence of any imaginary phonon modes indicates the dynamical stability of the structure. To verify the theoretical prediction, we then carefully synthesized the TaFeSb compound by the ball-milling and hot-pressing method. Rietveld refinement for the XRD pattern of the prepared specimen indicates that the single-phase TaFeSb with half-Heusler structure was successfully synthesized. The obtained lattice parameter by Rietveld refinement is ~0.5938 nm (Fig. 2c). The selected area electron diffraction (SAED) pattern for the prepared specimen viewed along the [111] direction is shown in Fig. 2d. The three-fold symmetry of the $F\bar 43{\mathrm{m}}$ cubic structure along the [111] direction is well demonstrated by the SAED pattern and the corresponding atomic-resolution image (as shown in Supplementary Fig. 1). The lattice parameter (~0.59 nm) observed by STEM is consistent with the XRD results.

### Electronic properties of TaFeSb-based half-Heuslers

**Fig. 3:**
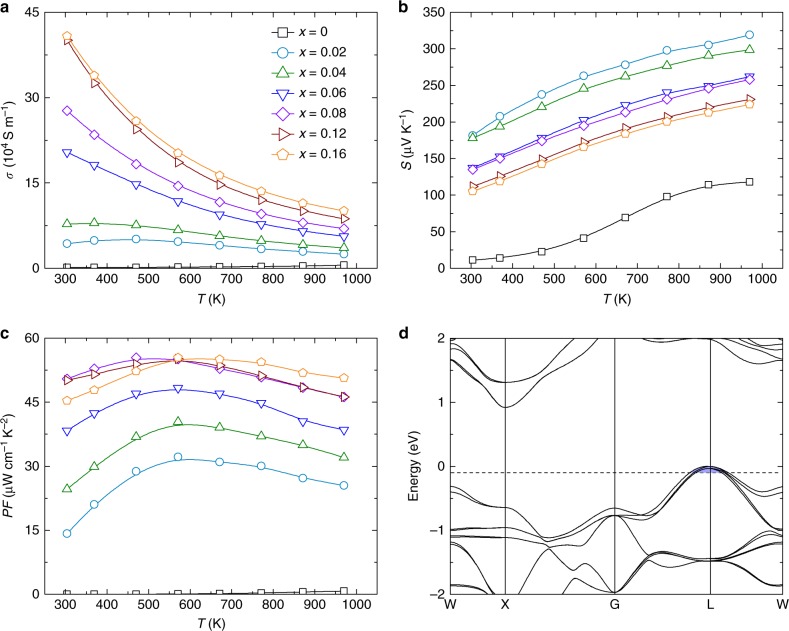
Electronic properties of TaFeSb-based half-Heuslers. Temperature-dependent **a** electrical conductivity, **b** Seebeck coefficient, and **c** power factor of Ta_1-*x*_Ti_*x*_FeSb (*x* = 0, 0.02, 0.04, 0.06, 0.08, 0.12, and 0.16). **d** Band structure of TaFeSb

Optimization of the thermoelectric performance of TaFeSb-based half-Heuslers was realized by tuning the carrier concentration via Ti-doping. In our work, Ta_1-*x*_Ti_*x*_FeSb (*x* = 0, 0.02, 0.04, 0.06, 0.08, 0.10, 0.12, 0.14, 0.16, and 0.18) samples were prepared by the ball-milling and hot-pressing method. All of the samples are highly dense with an average grain size of ~250 nm (as shown in Supplementary Fig. 2) and they are single-phase with high crystallinity (as shown in Supplementary Fig. 3). For simplicity, the thermoelectric properties of only some of the Ta_1-*x*_Ti_*x*_FeSb specimens are shown in Fig. 3, while the complete results can be found in Supplementary Fig. 4. As shown in Fig. 3a, the electrical conductivity of the pristine TaFeSb is very low but it increases markedly with Ti-doping. Enhancement of the electrical conductivity should be attributed to the substantially increased hole concentration, as shown in Supplementary Fig. 5. It is ~5.26 × 10^20^ cm^−3^ for Ta_0.98_Ti_0.02_FeSb and ~2.03 × 10^21^ cm^−3^ for Ta_0.84_Ti_0.16_FeSb. This indicates that Ti is a very efficient dopant for supplying a high concentration of holes to TaFeSb, which is similar to the case of Ti-doping in NbFeSb^24,25^. Due to the increased hole concentration, the Seebeck coefficient of Ta_1-*x*_Ti_*x*_FeSb (with the exception of TaFeSb) is reduced gradually with increasing Ti concentration, as shown in Fig. 3b. It should be noted that the undoped TaFeSb is fully p-type over the whole temperature range and its Seebeck coefficient increases with temperature without showing any bipolar conduction despite its low hole concentration. This should be ascribed to the relatively large band gap of ~0.53 eV for TaFeSb, which is even larger than that of NbFeSb (~0.41 eV), as shown in Supplementary Fig. 6. Optimization of the holeconcentration substantially improves the power factor of TaFeSb-based half-Heuslers, as shown in Fig. 3c. The room-temperature power factor is ~45 μW cm^−1^ K^−2^ for Ta_0.84_Ti_0.16_FeSb and its peak power factor can be as large as ~56 μW cm^−1^K^−2^, thus leading to its high average power factor of ~52 μW cm^−1^K^−2^ in the temperature range between 300 and 973 K. Such a high power factor outperforms most of the other state-of-the-art half-Heusler compounds, e.g., (Ti/Zr/Hf)CoSb^26–28^, (Ti/Zr/Hf)NiSn^29–33^, ZrNiPb^34^, and NbCoSn^35,36^. The origin of the high electronic thermoelectric performance of TaFeSb-based half-Heuslers can be partially ascribed to the unique electronic band structure, as shown in Fig. 3d. The valence band maximum locates at the L-point (with a carrier pocket degeneracy of four), where two valence bands (with an orbital degeneracy of two) effectively converge and thus leading to a very high valley degeneracy (*N*_v_) of eight (as shown in Supplementary Fig. 7). The thermoelectric figure of merit *ZT* is closely correlated with the materials’ parameter *B*, where $B \propto N_{\mathrm{V}}\mu \left( {m^ \ast } \right)^{3/2}/\kappa _{{\mathrm{lat}}}$ (*μ* is the mobility and m^*^ is the effective mass)^1,37^. Therefore, the high band degeneracy of TaFeSb is highly beneficial for realizing the large power factor.

### Thermal conductivities of TaFeSb-based half-Heuslers

Comparison of the thermal conductivity between the undoped NbFeSb and TaFeSb is shown in Supplementary Fig. 8. The room-temperature thermal conductivity is ~14 W m^−1^K^−1^ for NbFeSb and it is only ~8.8 W m^−1^K^−1^ for TaFeSb. To understand the difference in lattice thermal conductivity, phonon dispersion relations for NbFeSb and TaFeSb were calculated as shown in Supplementary Fig. 9. TaFeSb has overall lower phonon frequencies than those of NbFeSb. The acoustic phonon velocity is lower in TaFeSb compared to NbFeSb. Usually, the phonon velocity can simply be approximated by the low frequency sound velocity^38^. The measured sound velocity is 2907 m s^−1^ for TaFeSb but is 3473 m s^−1^ for NbFeSb (as shown in Supplementary Fig. 10). According to the kinetic theory, the lattice thermal conductivity can be expressed as $\kappa _{{\mathrm{lat}}}{\mathrm{ = }}\frac{1}{3}C_{\mathrm{v}}v_{{\mathrm{ph}}}l$, where *C*_v_ is the heat capacity per unit volume, *v*_ph_ is the phonon velocity, and *l* is the phonon mean free path^39^. Therefore, the lower phonon velocity of TaFeSb can account for its lower thermal conductivity compared to NbFeSb.

**Fig. 4:**
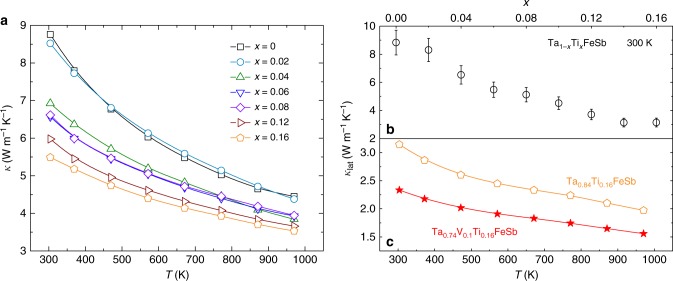
Thermal conductivity of TaFeSb-based half-Heuslers. **a** Temperature-dependent thermal conductivity of Ta_1-*x*_Ti_*x*_FeSb, **b** composition-dependent room-temperature lattice thermal conductivity of Ta_1-*x*_Ti_*x*_FeSb, and **c** comparison of lattice thermal conductivity between Ta_0.84_Ti_0.16_FeSb and Ta_0.74_V_0.1_Ti_0.16_FeSb

Although the electrical conductivity of TaFeSb has been substantially increased by Ti-doping (which effectively increases the electronic thermal conductivity via the Wiedemann-Franz law *κ*_ele_=*LσT*, where *L* is the Lorenz constant), the total thermal conductivity of Ta_1-*x*_Ti_*x*_FeSb demonstrates a noticeable reduction with increasing Ti concentration (as shown in Fig. 4a). The composition-dependent lattice thermal conductivity of Ta_1-*x*_Ti_*x*_FeSb is calculated and shown in Fig. 4b. It demonstrates a monotonic reduction with respect to Ti concentration. The room-temperature lattice thermal conductivity is ~8.8 W m^−1^K^−1^ for TaFeSb but is only ~3.1 W m^−1^K^−1^ for Ta_0.84_Ti_0.16_FeSb, a reduction of ~65%. Such a significant phonon scattering should be ascribed mainly to the presence of point defects induced by Ti-doping at the Ta site. The substantial difference in atomic mass between Ti (~47.87) and Ta (~180.95) leads to the strong scattering that effectively disrupts the phonon propagation. By alloying the Ta_0.84_Ti_0.16_FeSb composition with VFeSb, the lattice thermal conductivity can be further reduced, as shown in Fig. 4c and Supplementary Fig. 11e. The room-temperature lattice thermal conductivity is ~3.1 W m^−1^ K^−1^ for Ta_0.84_Ti_0.16_FeSb and is only ~2.3 W m^−1^ K^−1^ for Ta_0.74_V_0.1_Ti_0.16_FeSb, a decrease of ~26%.

### *ZT* and energy conversion efficiency of TaFeSb-based half-Heuslers

**Fig. 5:**
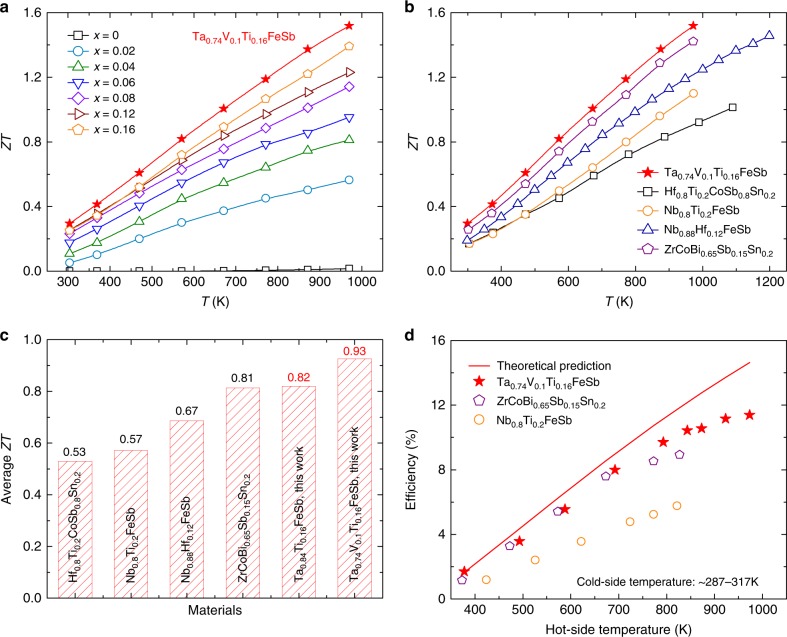
Dimensionless thermoelectric figure of merit of TaFeSb-based half-Heuslers. **a** Temperature-dependent *ZT* of Ta_1-*x*_Ti_*x*_FeSb, **b** comparison of the *ZT* between Ta_0.74_V_0.1_Ti_0.16_FeSb and the other state-of-the-art p-type half-Heuslers^24–27^, **c** comparison of the average *ZT* (in the range between 300 and 973 K) between TaFeSb-based half-Heuslers and the other state-of-the-art p-type half-Heuslers, and **d** the measured hot-side temperature-dependent heat-to-electricity conversion efficiency of Ta_0.74_V_0.1_Ti_0.16_FeSb as compared with state-of-the-art p-type half-Heuslers ZrCoBi_0.65_Sb_0.15_Sn_0.2_^40^ and Nb_0.8_Ti_0.2_FeSb^25^. The cold-side temperature is fixed at 300 K for the calculation of efficiency (solid line)

Benefitting from the simultaneous enhancement in power factor and reduction in thermal conductivity, the thermoelectric figure of merit of TaFeSb-based half-Heuslers can be effectively improved after Ti-doping. The thermoelectric performance of Ta_1-*x*_Ti_*x*_FeSb increases with the Ti concentration and Ta_0.84_Ti_0.16_FeSb achieves a high peak *ZT* of ~1.39 at 973 K (as shown in Fig. 5a). As mentioned earlier, by alloying Ta_0.84_Ti_0.16_FeSb with VFeSb, it is possible to further reduce the thermal conductivity and the power factor remains similar when V concentration is less than 15 at.% (as shown in Supplementary Fig. 11c). Therefore, the thermoelectric performance of TaFeSb-based half-Heuslers can be further improved and Ta_0.74_V_0.1_Ti_0.16_FeSb achieves a peak *ZT* of ~1.52 at 973 K. To demonstrate the repeatability of our results, Ta_0.74_V_0.1_Ti_0.16_FeSb has been prepared in five different batches and the thermoelectric properties are quite similar (as shown in Supplementary Fig. 12). The thermal stability of Ta_0.74_V_0.1_Ti_0.16_FeSb was further characterized by repeatedly measuring the same specimen five times and the thermoelectric properties are also comparable (as shown in Supplementary Fig. 13). This indicates that the high thermoelectric performance of Ta_0.74_V_0.1_Ti_0.16_FeSb is very stable and repeatable. Comparison of the thermoelectric performance between the TaFeSb-based half-Heuslers with the other p-type state-of-the-art half-Heusler compounds^24–27^ is shown in Fig. 5b. Clearly, Ta_0.74_V_0.1_Ti_0.16_FeSb outperforms all of the other half-Heuslers over the whole temperature range between 300 and 973 K. The average *ZT* is further calculated by the integration method (with a fourth-order polynomial for fitting the *ZT* curve) between 300 and 973 K and comparison of the average *ZT* is shown in Fig. 5c. The average *ZT*s are ~0.53 for Hf_0.8_Ti_0.2_CoSb_0.8_Sn_0.2_^26^, ~0.57 for Nb_0.8_Ti_0.2_FeSb^25^, ~0.67 for Nb_0.88_Hf_0.12_FeSb^24^, and ~0.81 for ZrCoBi_0.65_Sb_0.15_Sn_0.2_^40^, all of which are lower than ~0.82 for Ta_0.84_Ti_0.16_FeSb and ~0.93 for Ta_0.74_V_0.1_Ti_0.16_FeSb. The high thermoelectric performance of Ta_0.74_V_0.1_Ti_0.16_FeSb was further verified by the heat-to-electricity conversion efficiency measurement. As shown in Fig. 5d, the measured peak efficiency of Ta_0.74_V_0.1_Ti_0.16_FeSb can be as large as ~11.4% at the cold-side temperature of 317 K and hot-side temperature of 973 K. This is a record high efficiency for a single leg based on half-Heusler thermoelectric materials. Our results demonstrate that the TaFeSb-based half-Heuslers are quite promising for thermoelectric power generation.

## Discussion

In summary, thermodynamic stability analysis was performed for the V^1^-VIII-V^2^ family (with V^1^ = V, Nb, and Ta; VIII = Fe, Ru, and Os; and V^2^ = As, Sb, and Bi) of half-Heuslers. We predicted that six compounds (VRuAs, NbRuAs, TaRuAs, TaFeSb, NbOsSb, and TaOsSb) in this family should be stable. To verify this prediction, the TaFeSb-based half-Heuslers were synthesized and their thermoelectric performance was optimized. Our results indicated that Ta_0.74_V_0.1_Ti_0.16_FeSb can achieve a peak *ZT* of ~1.52 at 973 K, outperforming all of the other state-of-the-art p-type half-Heusler materials. More importantly, the heat-to-electricity conversion efficiency was also measured for the TaFeSb-based half-Heuslers and the maximum efficiency can be as large as ~11.4% at the cold-side temperature of 317 K and hot-side temperature of 973 K. Our work demonstrated that the TaFeSb-based half-Heuslers are quite promising for thermoelectric power generation.

## Methods

### Synthesis

TaFeSb-based half-Heuslers were prepared by a two-step ball-milling and hot-pressing method. In the first step, Ta turnings (99.98%; Atlantic Metals & Alloys) and Sb ingots (99.999%; Alfa Aesar) were loaded into a stainless-steel jar under an argon atmosphere in a glove box and ball-milled for 15 h. Afterward, Fe granules (99.8%; Alfa Aesar), Ti granules (99.99%; Alfa Aesar), and V pieces (99.7%; Alfa Aesar) were added for another 20 h ball milling. The ball-milling process was conducted using a SPEX 8000 M Mixer/Mill. The ball-milled powders were compacted to a disk by a direct-current-induced hot-pressing at about 1123 K for 2 min and under the pressure of ~80 MPa.

### Thermoelectric properties measurement

The Seebeck coefficient and electrical conductivity were simultaneously obtained by a commercial (ZEM-3, ULVAC) system in helium atmosphere. The thermal conductivity *κ* *=* *DC*_p_*ρ* was calculated from the thermal diffusivity *D* (LFA 457, Netzsch), specific heat *C*_*p*_ (DSC 404 C; Netzsch), and mass density *ρ* (Archimedes’ kit). Hall carrier concentrations (*n*_H_) were measured using a commercial system (PPMS Dynacool, Quantum Design) with a magnetic field of ±3 T and an electrical current of 8 mA.

### Efficiency measurement

The thermoelectric material (referenced as leg in the following discussion) was polished to the size of 2.6 × 2.7 mm^2^ in cross-section and ~11 mm in length. The cold-side temperature was maintained at around 287–317 K by water circulation. The experiments were conducted under high vacuum (below 10^−6^ mbar) to reduce the parasitic conduction and convection losses. To measure conversion efficiency (*η*), the input power from the hot side (*Q*_in_) and the generated power (*P*) from the thermoelectric leg were measured at the same time. The direct measurement of *Q*_in_ is a greatly challenging due to the heavy heat loss at high temperature. According to Fourier’s law, a bulk polycrystalline graphite with measured geometry and thermal conductivity was placed below the cold-side to measure the heat flow out of the cold-side (*Q*_out_). The thermal conductivity of the bulk polycrystalline graphite was confirmed by the method described above in the discussion on thermoelectric properties measurement. In order to measure temperature differences of the leg and graphite bulk, K-type thermocouples were embedded at the interfaces. It should be noted that the hot-side temperature of graphite can be regarded as the cold-side temperature of the leg if the setup is working under a large pressure. The total *Q*_in_ equals the sum of *Q*_out_, *P*, and radiation loss from the leg (*Q*_rad_). Therefore, the conversion efficiency (*η*) can be written as the following

4 $$\eta {\mathrm{ = }}\frac{P}{{Q_{{\mathrm{in}}}}} = \frac{P}{{Q_{{\mathrm{out}}} + P + Q_{{\mathrm{rad}}}}}.$$

Since *Q*_rad_ cannot be directly measured, in real measurement *Q*_in_ is composed of *Q*_out_ and *P*, which leads to the measurement error of *η*. By tuning the current in the circuit, a series of *Q*_in_, *P* can be measured at the same time. Therefore, both maximum *η* and *P* can be found. The estimated radiation loss based on the finite difference method is about 1–6% of the total heat flow in.

For *p*-type thermoelectrics, the measured open circuit voltage should be smaller since the Seebeck coefficient of copper is a positive number, which can be given by a following empirical function^41^

5 $$\begin{array}{l}S_{{\mathrm{Cu}}}\left( T \right) = 0.041T\left[ {{\mathrm{exp}}\left( { - \frac{T}{{93}}} \right) + 0.123 - \frac{{0.442}}{{1 + (T/172.4)^3}}} \right]+ 0.804,\\ 70{\mathrm{K}} < T < 1000{\mathrm{K}}{\mathrm{.}}\end{array}$$

The Seebeck coefficient of copper is ~6 μV K^−1^ at 973 K, and the measured output power at 973 K is underestimated by about 5-6%.

### Microstructural characterization

Phase identification was carried out by X-ray diffraction (XRD) on a PANalytical multipurpose diffractometer with an X’Celerator detector (PANalytical X’Pert Pro). The morphology and microstructures were characterized by a field emission scanning electron microscope (FESEM, LEO 1525). Selected area electron diffraction pattern and HAADF-STEM images were obtained by a JEM-ARM 200F TEM operated at 200 kV.

### Bandgap measurement

Fourier transform infrared spectroscopy (FTIR) was performed to derive the optical band gap based on the Kramers-Kronig analysis of the reflectance. The FTIR was conducted using a Nicolet iS50 FT-IR spectrometer with a Spectra-Tech model 500 series variable angle specular reflectance accessory at room temperature.

### Sound velocity measurement

Sound velocity measurements were carried out using a RITEC Advanced Ultrasonic Measurement System RAM-5000. The system realizes the pulse-echo method of time propagation measurements with an accuracy of about 10^−3^ µs. To generate longitudinal (L) and shear (S) ultrasonic bulk waves, Olympus transducers V129-RM (10 MHz) and V157-RM (5 MHz) were used. Propylene glycol and SWC (both from Olympus) were used as couplant materials for the L and S modes, respectively. Thickness measurements were carried out using a Mitutoyo ID-HO530 device. All data were obtained at 300 K.

### Theoretical calculations

We searched for stable V^1^-VIII-V^2^ half-Heusler crystal structures up to 18 atoms per unit cell using the global optimization particle swarm method as implemented in the CALYPSO code^42,43^. At least 2000 structures were calculated during each search. The key feature of the CALYPSO method is the ability to rapidly identify ground-state structures with knowledge only of the chemical composition. Details of the algorithm and applications have been discussed elsewhere^44–46^. The first-principles calculations of the formation energy were performed using the projector-augmented wave (PAW) method as implemented in the VASP code^47,48^. The electronic structures were obtained with the general potential linearized augmented plane wave (LAPW) method as implemented in the WIEN2k code^49^. This enabled us to realize structure optimization and hybrid functional calculations using VASP, which is time consuming and has the potential compatibility issue (with CALYPSO) in WIEN2K, as well as allowing the convenience of the later transport calculations using WIEN2K. The Perdew, Burke, and Ernzerhof (PBE-GGA) type functional was used for the structural optimization within VASP^50^. Because of the sensitivity of thermodynamic stability, the 3*p*^6^3*d*^3^4*s*^2^ (V), 4*p*^6^4*d*^4^5*s*^1^ (Nb), 5*p*^6^5*d*^3^6*s*^2^ (Ta), 3*p*^6^3*d*^6^4*s*^2^ (Fe), 4*p*^6^4*d*^7^5*s*^1^ (Ru), 5*p*^6^5*d*^6^6*s*^2^ (Os), 4*s*^2^4*p*^3^ (As), 5*s*^2^5*p*^3^ (Sb), and 5*d*^10^6*s*^2^6*p*^3^ (Bi) states were treated as valence electrons of PAW pseudopotentials. These were used with kinetic energy cutoffs of 520 eV. We conducted tests to ensure that this was adequate. The k-point meshes for sampling the Brillouin zone were at a grid spacing of 2*π* × 0.032 Å or better, including the hybrid functional calculations. Harmonic phonons were obtained with real space supercells using the PHONOPY code^51^.

Band structure and density of states (DOS) calculations were conducted by using the modified Becke–Johnson (mBJ) potential of Tran and Blaha^52,53^. This mBJ potential gives band gaps in agreement with experiment for a wide variety of simple semiconductors and insulators^54,55^. In the present case, our calculated band gap of 0.9 eV is larger than the experimental value, perhaps due to site disorder in the half-Heusler structure. We also cross-checked the band gaps using the hybrid Heyd–Scuseria–Ernzerhof (HSE06) functional^56^, which also supports a larger band gap. Spin–orbit coupling (SOC) was included in electronic structure calculations, as the spin–orbit splitting may be very large for heavy elements. We used LAPW sphere radii of 2.5 bohr for TaFeSb. A basis set cutoff, *K*_max_, determined by the criterion *R*_min_*K*_max_ = 9.0, was used. Here, *R*_min_ denotes the minimum atomic radius of elements.

## Supplementary information

Supplementary Information

## Data Availability

The data that support the findings of this study are available from the corresponding author upon reasonable request.
