# Supplementary material for: Discovery of TaFeSb-based half-Heuslers with high thermoelectric performance
Source: Nat Commun. 2019 Jan 17;10:270. doi: 10.1038/s41467-018-08223-5 (PMC6336844; doi:10.1038/s41467-018-08223-5)
Supplement: Supplementary file 1 — Supplementary Information [file 41467_2018_8223_MOESM1_ESM.pdf]

Supplementary Information

Discovery of TaFeSb-based half-Heuslers with high thermoelectric performance

Hangtian Zhu[†1], Jun Mao[†1], Yuwei Li[2], Jifeng Sun[2], Yumei Wang[3], Qing Zhu[1], Guannan Li[4], Qichen Song[5], Jiawei Zhou[5], Yuhao Fu[2], Ran He[6], Tian Tong[7], Zihang Liu[1], Wuyang Ren[1,8], Li You[1,9], Zhiming Wang[8], Jun Luo[9], Andrei Sotnikov[6], Jiming Bao[7], Kornelius Nielszch[6], Gang Chen[5], David J. Singh[2]*, and Zhifeng Ren[1]*

[1] *Department of Physics and Texas Center for Superconductivity, University of Houston, Houston, TX 77204, U.S.A.*

[2] *Department of Physics and Astronomy, University of Missouri, Columbia, MO 65211, U.S.A.*

[3] *Beijing National Laboratory for Condensed Matter Physics, Institute of Physics, Chinese Academy of Sciences, P.O. Box 603, Beijing 100190, China*

[4] *Department of Materials and Energy, Southwest University, Chongqing 400715, China*

[5] *Department of Mechanical Engineering, Massachusetts Institute of Technology, Cambridge, MA 02139, U.S.A.*

[6] *Institute for Metallic Materials, IFW-Dresden, Dresden 01069, Germany*

[7] *Department of Electrical and Computer Engineering, University of Houston, Houston, TX 77204, United States*

[8] *Institute of Fundamental and Frontier Sciences, University of Electronic Science and Technology of China, Chengdu 610054, China*

[9] *School of Materials Science and Engineering, Shanghai University, Shanghai 200444, China*

[†] These authors contribute equally to this work.

[*]Author to whom correspondence should be addressed: singhdj@missouri.edu; zren@uh.edu.

Supplementary Figures

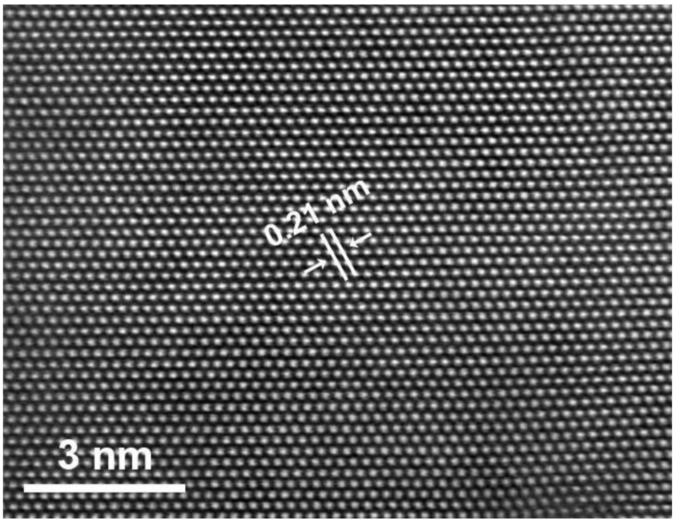

Supplementary Figure 1. STEM image of the prepared TaFeSb half-Heusler. The $\left(2\bar{2}0\right)$ plane with a spacing of ~0.21 nm has been verified.

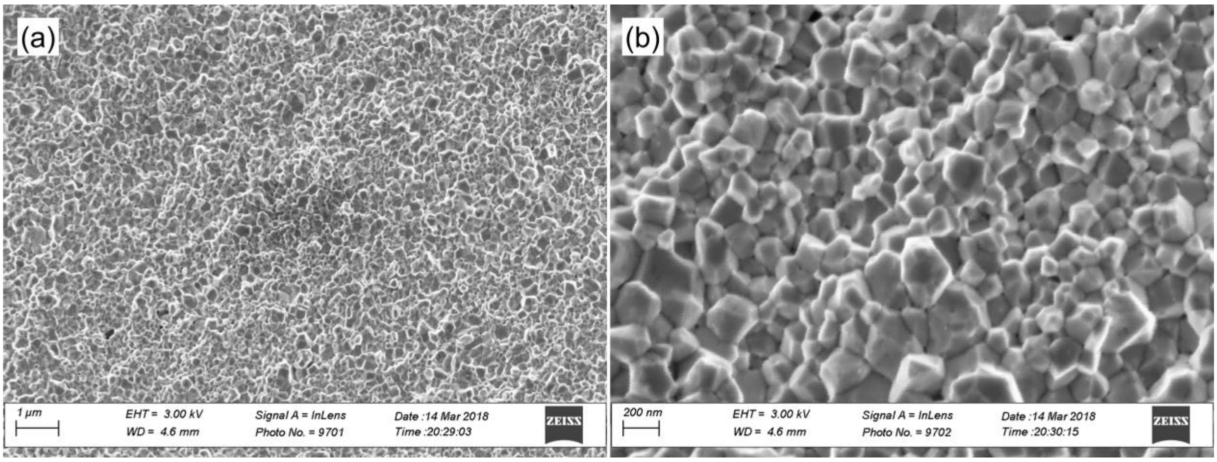

Supplementary Figure 2. Microstructures of the pristine TaFeSb half-Heusler. (a) and (b) Scanning electron microscopy images.

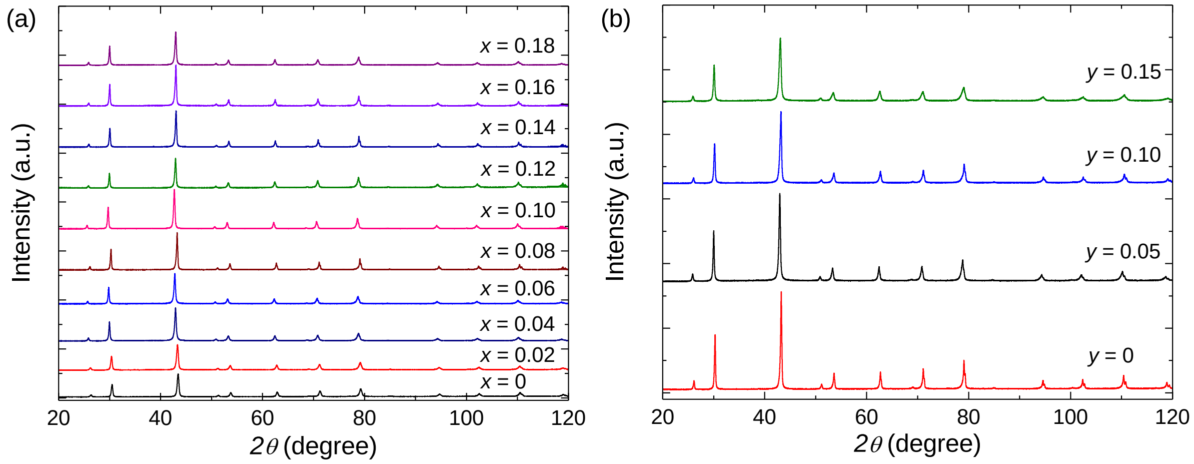

Supplementary Figure 3. Phase composition of TaFeSb-based half-Heuslers. XRD patterns of the prepared (a) $Ta_{1-x}Ti_xFeSb$ (x = 0, 0.02, 0.04, 0.06, 0.08, 0.10, 0.12, 0.14, 0.16, and 0.18) and (b) $Ta_{0.84-y}V_yTi_{0.16}FeSb$ (y = 0, 0.05, 0.10, and 0.15).

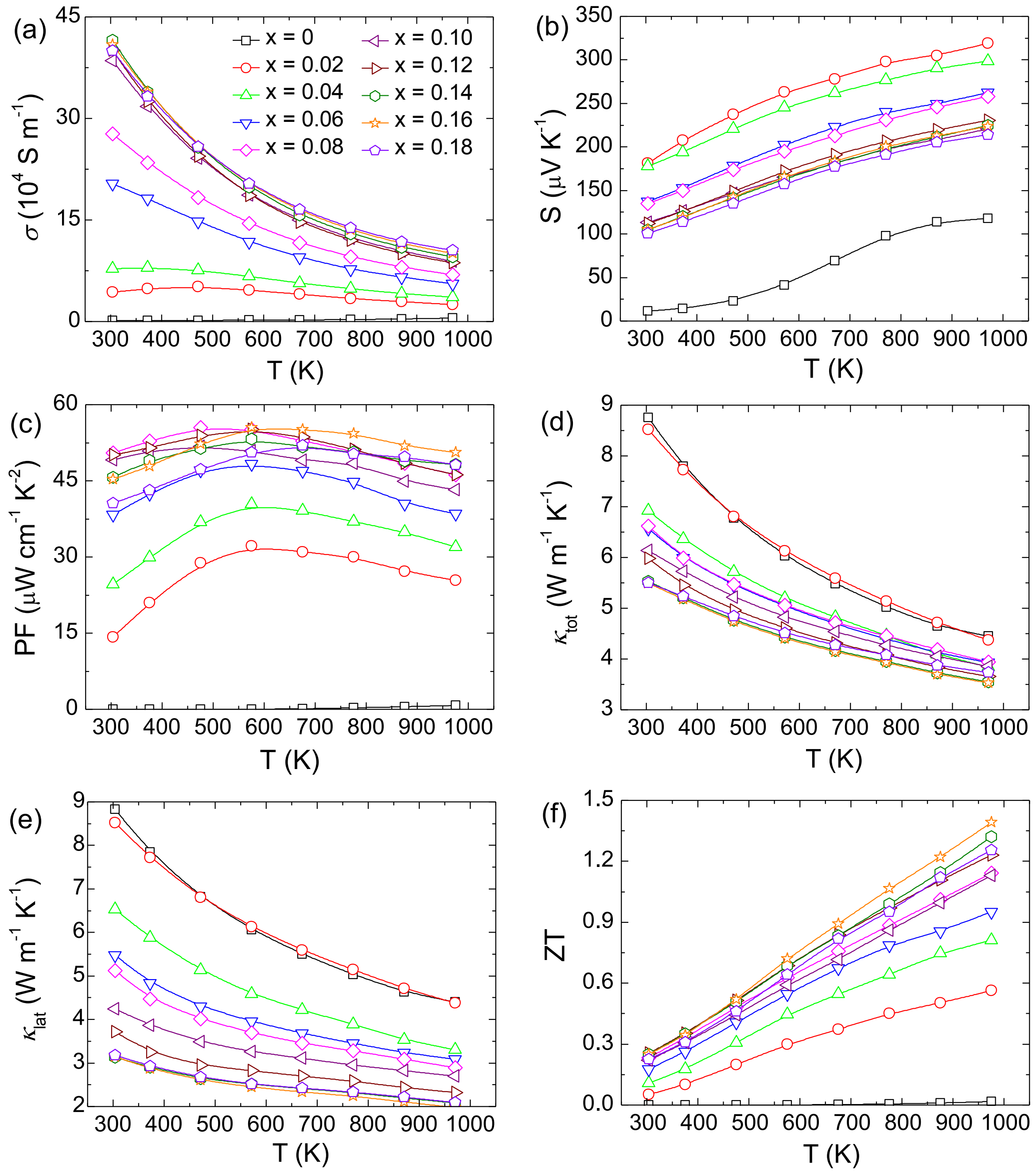

Supplementary Figure 4. Thermoelectric properties of $Ta_{1-x}Ti_xFeSb$ (x = 0, 0.02, 0.04, 0.06, 0.08, 0.10, 0.12, 0.14, 0.16, and 0.18). (a) Electrical conductivity, (b) Seebeck coefficient, (c) power factor, (d) total thermal conductivity, (e) lattice thermal conductivity, and (f) *ZT*.

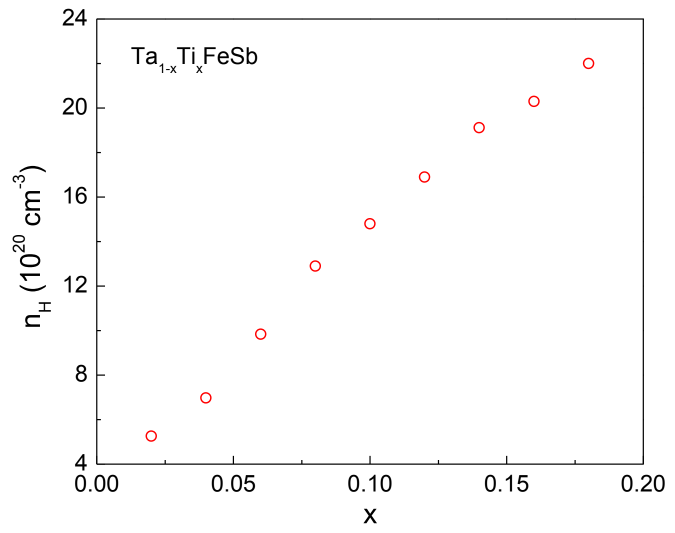

Supplementary Figure 5. Composition-dependent room-temperature hole concentration.

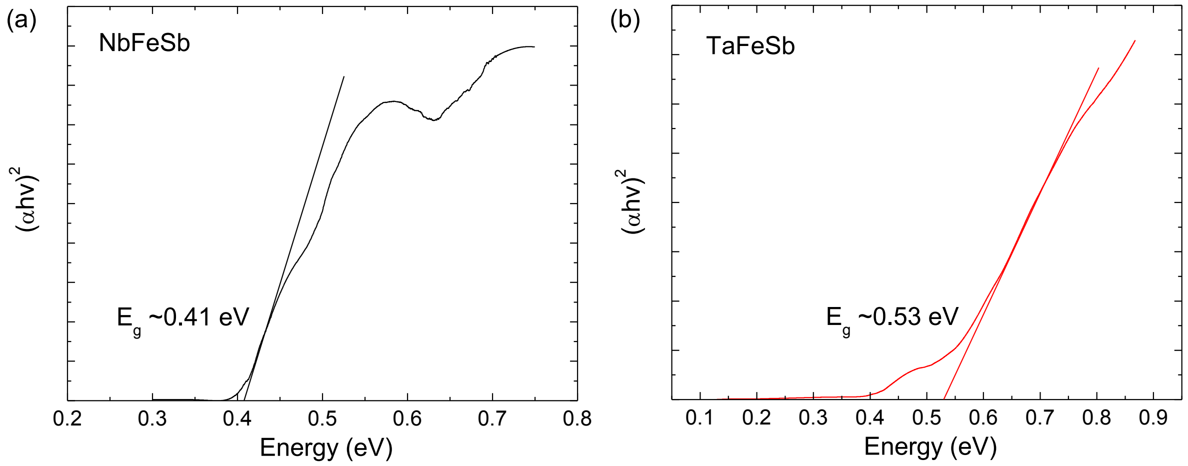

Supplementary Figure 6. Measured bandgap of the pristine (a) NbFeSb and (b) TaFeSb.

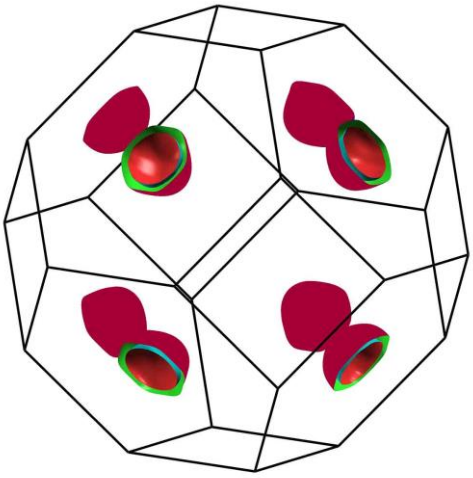

Supplementary Figure 7. The iso-energy carrier pockets of TaFeSb at 0.1 eV below the valence band maximum.

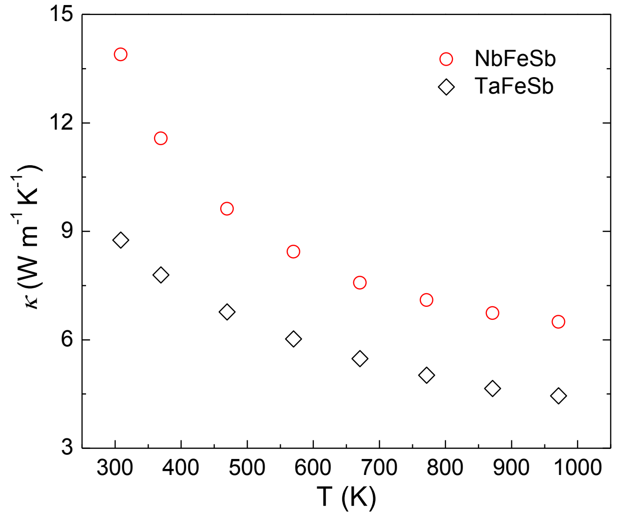

Supplementary Figure 8. The thermal conductivity of the undoped NbFeSb and TaFeSb. The samples are both prepared by the identical ball-milling and hot-pressing method.

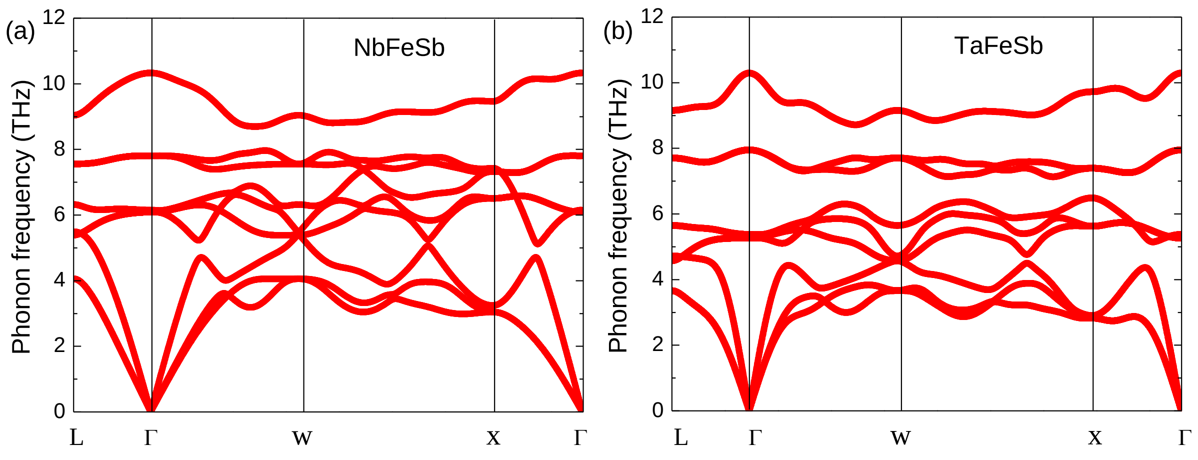

Supplementary Figure 9. Comparison of the phonon dispersion between (a) NbFeSb and (b) TaFeSb.

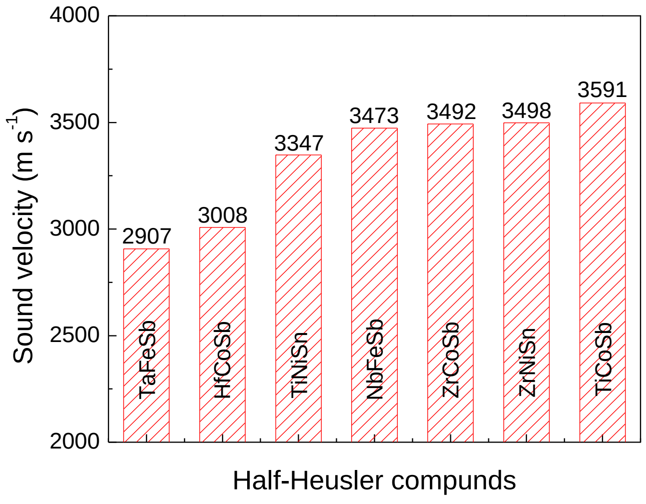

Supplementary Figure 10. Comparison of sound velocity between several half-Heusler compounds.

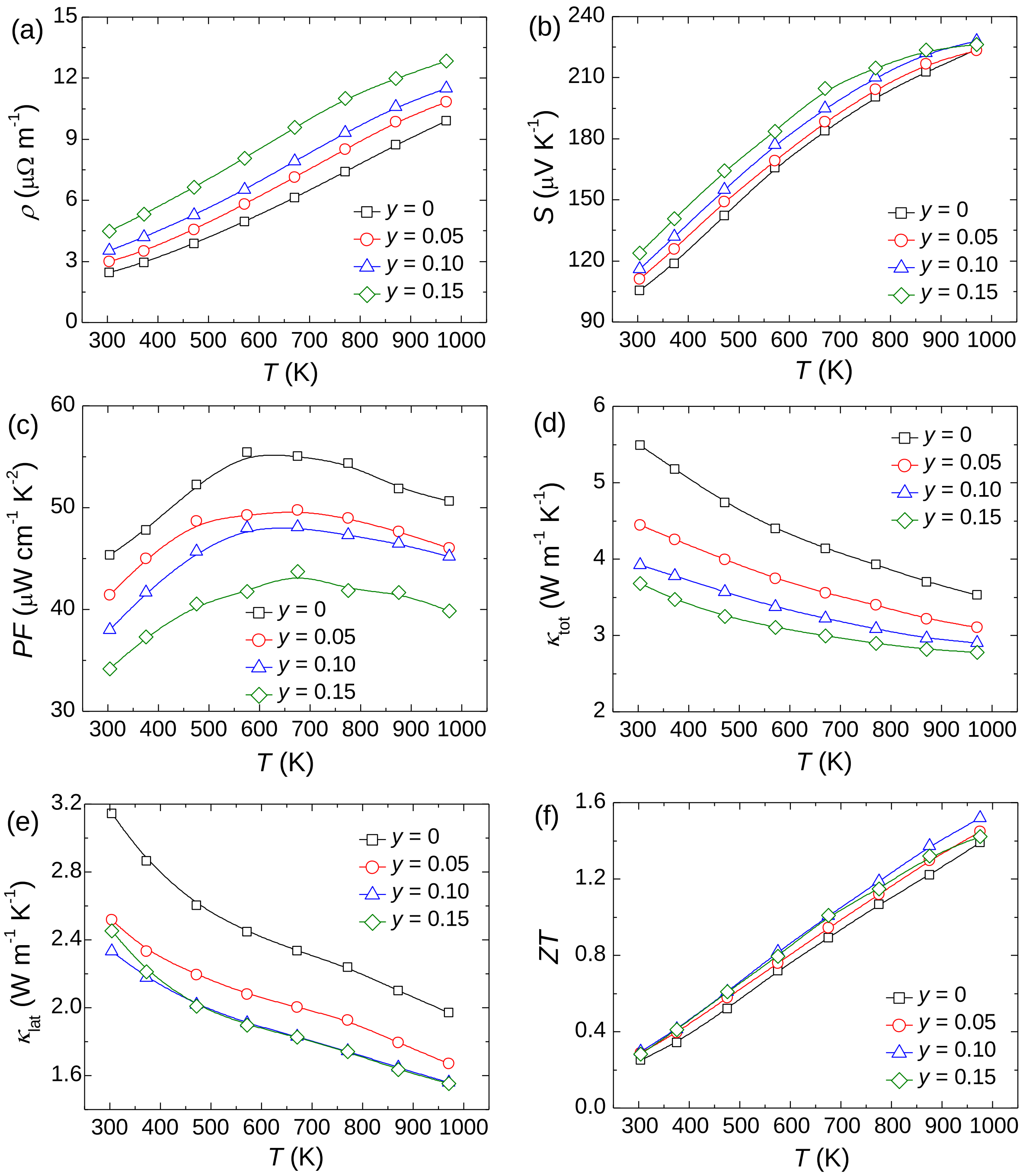

Supplementary Figure 11. Thermoelectric properties of $Ta_{0.84-y}V_yTi_{0.16}FeSb$ (y = 0, 0.05, 0.10, and 0.015). (a) Electrical resistivity, (b) Seebeck coefficient, (c) power factor, (d) total thermal conductivity, (e) lattice thermal conductivity, and (f) *ZT*.

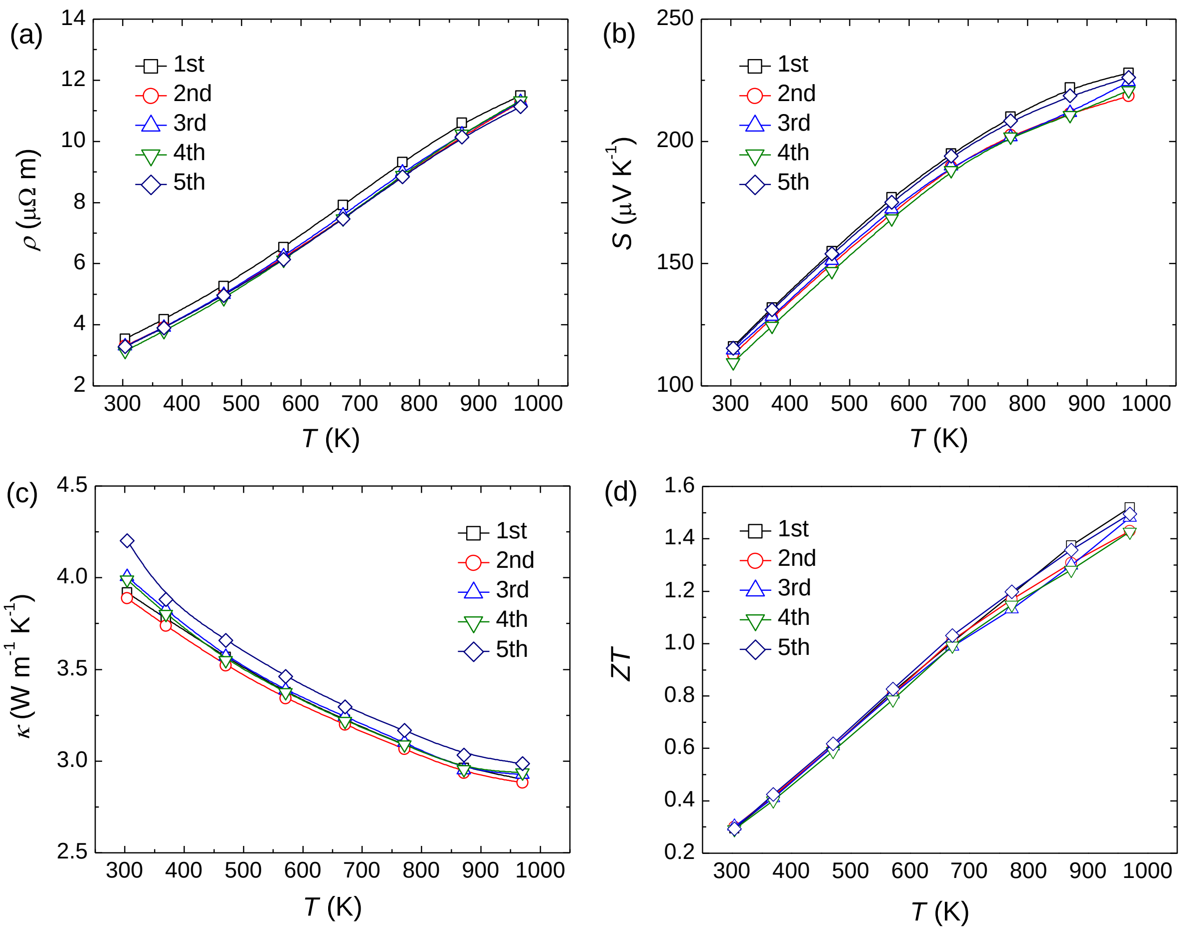

Supplementary Figure 12. Reproducibility of the thermoelectric properties of $Ta_{0.74}V_{0.1}Ti_{0.16}FeSb$. Five different batches of $Ta_{0.74}V_{0.1}Ti_{0.16}FeSb$ were prepared and the thermoelectric properties of the specimens were then characterized. (a) Electrical resistivity, (b) Seebeck coefficient, (c) thermal conductivity, and (d) *ZT*.

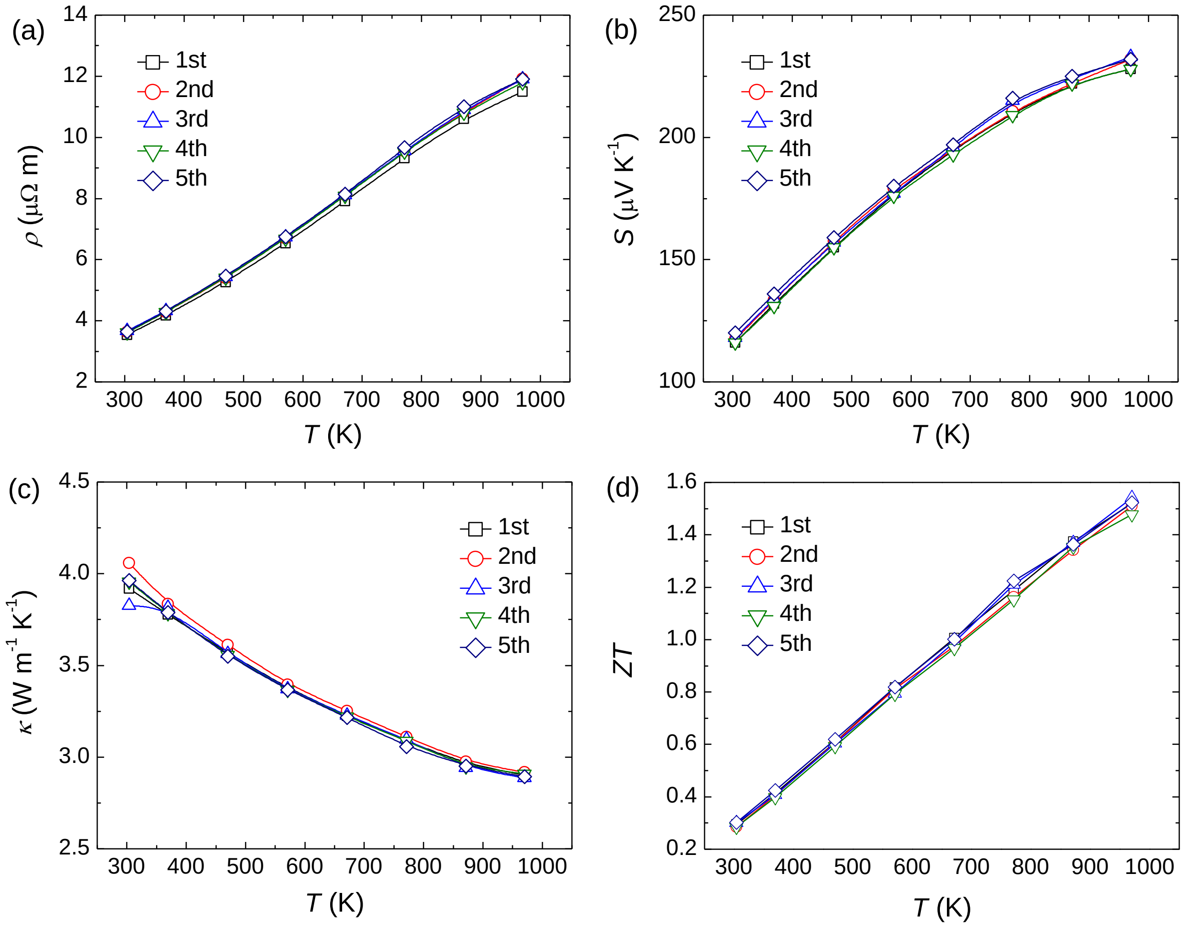

Supplementary Figure 13. Thermal stability of $Ta_{0.74}V_{0.1}Ti_{0.16}FeSb$. Thermoelectric properties of the same $Ta_{0.74}V_{0.1}Ti_{0.16}FeSb$ specimen measured five times. (a) Electrical resistivity, (b) Seebeck coefficient, (c) thermal conductivity, and (d) *ZT*.
